# Patterns of antimicrobial resistance in a surgical intensive care unit of a university hospital in Turkey

**DOI:** 10.1186/1471-2334-6-155

**Published:** 2006-10-25

**Authors:** Aysen Bayram, Iclal Balci

**Affiliations:** 1 Gaziantep University Faculty of Medicine, Department of Microbiology and Clinical Microbiology, Gaziantep, Turkey

## Abstract

**Background:**

Several studies have reported higher rates of antimicrobial resistance among isolates from intensive care units than among isolates from general patient-care areas. The aims of this study were to review the pathogens associated with nosocomial infections in a surgical intensive care unit of a university hospital in Turkey and to summarize rates of antimicrobial resistance in the most common pathogens. The survey was conducted over a period of twelve months in a tertiary-care teaching hospital located in the south-eastern part of Turkey, Gaziantep. A total of 871 clinical specimens from 615 adult patients were collected. From 871 clinical specimens 771 bacterial and fungal isolates were identified.

**Results:**

Most commonly isolated microorganisms were: *Pseudomonas aeruginosa *(20.3%), *Candida *species (15%) and *Staphylococcus aureus *(12.9%). Among the Gram-negative microorganisms *P. aeruginosa *were mostly resistant to third-generation cephalosporins (71.3–98.1%), while *Acinetobacter baumannii *were resistant in all cases to piperacillin, ceftazidime and ceftriaxone. Isolates of *S. aureus *were mostly resistant to penicillin, ampicillin, and methicillin (82–95%), whereas coagulase-negative staphylococci were 98.6% resistant to methicillin and in all cases resistant to ampicillin and tetracycline.

**Conclusion:**

In order to reduce the emergence and spread of antimicrobial-resistant pathogens in ICUs, monitoring and optimization of antimicrobial use in hospitals are strictly recommended. Therefore local resistance surveillance programs are of most value in developing appropriate therapeutic guidelines for specific infections and patient types.

## Background

Antimicrobial resistance among intensive care unit (ICU) pathogens is generally increasing, but variations do exist among different countries, probably due to individual antimicrobial use patterns. When new medical practices and alternative antimicrobials are introduced, changes in the dominant microbial etiologies may emerge prompting novel empiric selections. Appropriate therapy of ICU infections directed by local resistance data can have significant consequences for both patient and the healthcare system.

Data from National Nosocomial Infections Surveillance (NNIS) shows that from 1988 to 1995 the number of intensive care unit (ICU) beds at the hospitals has increased 17%, whereas total hospital bed capacity decreased slightly [1]. Patients receiving care in ICUs are at high risk for nosocomial infections. The emergence of antimicrobial-resistant pathogens in ICUs has made treating these infections very difficult and, in some cases, impossible. Intensive care unit patients are particularly susceptible to nosocomial infections due to underlying illnesses, suppressed immune systems and frequent use of invasive devices.

This study reviews the pathogens associated with nosocomial infections among ICU patients, summarizes rates of antimicrobial resistance in the most common pathogens and provides an overview of strategies to prevent the proliferation of antimicrobial-resistant microorganisms.

## Results

During the study period a total of 871 clinical specimens from 615 adult patients were collected. None of the patients were receiving immunosuppressive therapy. In 771 (88.5%) of cases the isolate recovered by culture was confirmed to be the etiologic agent of a nosocomial infection. The distribution of infections was examined by the major site of infection. Four major infection sites represented 91.8% of all reported infections; lower respiratory tract infections were most frequent (31.5%), followed by urinary tract infections (27.8%), bloodstream infections (23.1%) and surgical site infections (9.3%).

The percentages of most frequently isolated microorganisms in ICU were as follows: *P. aeruginosa *20.3%, *Candida *spp. 15%, *S. aureus *12.9%, *A. baumannii *9.6%, and coagulase-negative staphylococci 8.9%. In lower respiratory tract infections, *P. aeruginosa *(27.9%) was the most commonly isolated pathogen, which was followed by *S. aureus *(13.6%) and A. *baumannii *(13.2%). Fifty-seven percent of reported isolates from the urinary tract were aerobic Gram-negative bacilli, and in 16.3% of cases *Candida *spp. were isolated. Bloodstream infections were mostly caused *S. aureus *and coagulase-negative staphylococci with 16.8% and 16.3%, respectively, whereas *P. aeruginosa *species (14.6%) were also frequently isolated from blood. In surgical site infections *S. aureus *(23.6%) and *P. aeruginosa *(22.2%) were the most commonly isolated infectious agents (Table 1).

**Table 1:** Microorganisms reported from intensive care unit according to the site of infection.

Microorganism	Respiratory Tract		Urinary Tract		Bloodstream		Surgical Site		Other Sites^1^	
	n	%	n	%	n	%	n	%	n	%
*Pseudomonas aeruginosa*	68	27.9	30	13.9	26	14.6	16	22.2	17	26.9
*Staphylococcus aureus*	33	13.6	9	4.2	30	16.8	17	23.6	11	17.5
*Acinetobacter baumannii*	32	13.2	20	9.3	11	6.2	6	8.3	5	7.9
*Candida *spp.	31	12.8	35	16.3	30	16.8	10	13.9	10	15.8
CN^2 ^– staphylococci	16	6.6	10	4.6	29	16.3	10	13.9	4	6.4
*Klebsiella pneumoniae*	11	4.5	9	4.2	2	1.1	2	2.8	1	1.6
*Enterobacter *spp.	10	4.1	13	6.1	6	3.4	2	2.8	1	1.6
*Enterococcus *spp.	6	2.5	22	10.2	13	7.3	1	1.4	2	3.2
*Escherichia coli*	3	1.2	27	12.6	4	2.3	3	4.2	1	1.6
Others	33	13.6	40	18.6	27	15.2	5	6.9	11	17.5
Total	243	100	215	100	178	100	72	100	63	100

^1 ^Cerebrospinal fluid, pericardial fluid, peritonal fluid, synovial fluid, biopsy, oral swab, rectal and anal swabs

^2 ^Coagulase-negative

Each of the pathogens listed on Table 1 has demonstrated antimicrobial resistance to at least one, if not several, of the antimicrobial agents commonly used to treat infections caused by these pathogens. In general, Gram-positive microorganisms such as *S. aureus *and coagulase-negative staphylococci were commonly associated with bloodstream or surgical site infections. Examination of the rates of antimicrobial resistance among these pathogens showed that rates of methicillin-resistant *S. aureus *(MRSA) and methicillin-resistant coagulase-negative staphylococci were 82% and 98.6%, respectively (Table 2). Antimicrobial resistance profiles of MRSA and MSSA (methicillin-sensitive *S. aureus*) isolated from infections in ICU were shown in Table 3.

**Table 2:** Antimicrobial susceptibilities of Gram-positive microorganisms isolated from infections in ICU.

	Percentage (%) of isolates resistant to indicated drug by broth microdilution		
Antimicrobial	*S. aureus *n = 100	CN^1 ^– staphylococci n = 69	*Enterococcus *spp. n = 44

Penicillin G	95%	98.6%	84.1%
Ampicillin	95%	100%	77.3%
Amoxicillin/clavulanate	81%	88.4%	77.3%
Methicillin	82%	98.6%	50%
Vancomycin	0%	0%	0%
Clindamycin	72%	66.7%	86.4%
Erythromycin	86%	86.9%	86.4%
TMP/SMX^2^	39%	100%	75%
Ciprofloxacin	78%	37.7%	81.8%
Tetracycline	86%	100%	84.1%

^1 ^Coagulase-negative

^2 ^Trimethoprim/sulfamethoxazol

**Table 3:** Antimicrobial resistances of MRSA and MSSA isolated from infections in ICU.

Percentage (%) of isolates resistant to antimicrobial by broth microdilution		
Antimicrobial	MRSA^1 ^n = 82	MSSA^2 ^n = 18

Vancomycin	0%	0%
Clindamycin	73.2%	66.7%
Erythromycin	90.2%	66.7%
TMP/SMX^3^	42.7%	22.2%
Ciprofloxacin	79.3%	72.2%
Tetracycline	87.8%	77.7%

^1 ^Methicillin-resistant *S. aureus*

^2 ^Methicillin-susceptible *S. aureus*

^3 ^Trimethoprim/sulfamethoxazol

Gram-negative bacilli are frequently associated with nosocomial infections in ICU patients (Table 1). *Pseudomonas aeruginosa *showed high proportion of resistance against antipseudomonal penicillins (piperacillin 69.4%, ticarcillin 93%) and third-generation cephalosporins (ceftazidime 71.3%, cefotaxime 96.2%, ceftriaxone 98.1%). Isolates of *A. baumannii *were in all cases resistant to ceftriaxone and in most cases to aztreonam (94.6%) and ticarcillin (93.2%). Although *Enterobacter *species showed multiple resistance to most antimicrobials tested, they were uniformly susceptible to imipenem and ciprofloxacin. *Klebsiella pneumoniae *were usually resistant to multiple antimicrobials and hydrolized third-generation cephalosporins and aztreonam (72–88%) (Table 4).

**Table 4:** Antimicrobial susceptibilities of Gram-negative microorganisms isolated from infections in ICU.

Percentage (%) of isolates resistant to antimicrobial by broth microdilution					
Antimicrobial	*P. aeruginosa *n = 157	*A. baumannii *n = 74	*Enterobacter *spp n = 32	*K. pneumoniae *n = 25	*E. coli *n = 38

Amikacin	29.9%	71.6%	59.4%	16%	18.4%
Azteronam	77.1%	94.6%	81.2%	88%	86.8%
Piperacillin	69.4%	100%	71.8%	76%	68.4%
Ticarcillin	93%	93.2%	81.2%	64%	68.4%
Ceftriaxone	98.1%	100%	59.4%	84%	84.2%
Cefotaxime	96.2%	100%	59.4%	84%	84.2%
Ceftazidime	71.3%	100%	75%	88%	86.8%
Ciprofloxacin	59.2%	56.7%	0%	20%	39.5%
Imipenem	26.1%	63.5%	0%	12%	13.1%
Cefoperazone	93%	100%	62.5%	72%	86.8%
Gentamicin	48.4%	85.1%	81.2%	80%	78.9%

## Discussion

A number of factors contribute to the emergence of antimicrobial resistance in ICUs including the severity of patient illness, predisposition to nosocomial infections, cross-transmission of pathogens characteristic of critical care areas within the hospital, compromised membrane and skin barriers following the use of invasive devices, extended length of hospital stay, and the widespread use of prophylactic and therapeutic anti-infective agents [2,3].

The types of organisms that have emerged as most problematic for patients within the ICU include the members of the family *Enterobacteriaceae*, non-fermenters *(P. aeruginosa *and *Acinetobacter *spp.) oxacillin-resistant *S. aureus *and vancomycin-resistant enterococci. All of these resistance profiles have been documented in other regional and global ICU surveillance studies including Project Intensive Care Antimicrobial Resistance Epidemiology (ICARE, 1994–2000), the Meropenem Yearly Susceptibility Test Information Collection Program (MYSTIC, 1997–2000), the ICU Surveillance Study (ISS, 1990–1993, 1994–2000) and the SENTRY Program (Europe, 1997–1998) [3-8].

The rank order of pathogens recovered in SENTRY Antimicrobial Surveillance Program was *S. aureus *(24.1%), *P. aeruginosa *(12.2%), *E. coli *(10.1%), *Klebsiella *spp. (8.9%), *Enterococcus *spp. (7.2%), coagulase-negative staphylococci (7%) and *Enterobacter *spp. (7%) (5). In the MYSTIC Study, *P. aeruginosa *(33%) was the most frequent isolate, followed by *A. baumannii *(17.1%), *K. pneumoniae *(12.1%), *E. coli *(10.5%) and *E. cloacae *(7.9%) (Garcia-Rodriguez and Jones, 2002). In this study *P. aeruginosa *(20.3%) was the most commonly isolated pathogen in ICU, which was followed by *Candida *spp. (15%), *S. aureus *(12.9%), *A. baumannii *(9.6%), and coagulase-negative staphylococci (8.9%).

Patients in the ICU are more likely than others to be colonized or infected with an antimicrobial-resistant pathogen, therefore the rates of resistance are significantly higher in patients cared for in the ICU than in non-ICU patients [9-14].

Examination of Gram-positive microorganisms, such as *S. aureus *and coagulase-negative staphylococci shows that rates of methicillin-resistant isolates in ICU have increased steadily over the past decade [13-15]. In our study 82% of *S. aureus *strains and 98.6% of coagulase-negative staphylococci were resistant to methicillin.

Gram-negative bacilli are frequently associated with nosocomial infections in ICUs. Data from a multicenter Intensive Care Unit Surveillance Study (ISS) in the United States demonstrated that resistance to antipseudomonal agents among ICU isolates of *P. aeruginosa*, especially fluoroquinolones, was increasing [16]. The isolates *of P. aeruginosa *in this study were resistant 71.4 to 98% to third generation cephalosporins, and 69.4 to 93% to antipseudomonal penicillins. The relatively high susceptibility of Gram-negative bacilli to fluoroquinolones in this study can be attributed to antimicrobial prescribing protocol used in our ICU, which is preferably directed to broad-spectrum antibiotics.

In recent years *Acinetobacter *spp. have emerged as important pathogens of ICUs, most of them being resistant to ampicillin, carbenicillin, cefotaxime, chloramphenicol, and gentamicin [17,18]. In our study, all *A. baumannii *isolates were resistant to third generation cephalosporins, and most of them were resistant to aztreonam, ticarcillin, and gentamicin with 94.6%, 93.2%, and 85.1%, respectively.

*Enterobacter *species are resistant to first-generation cephalosporins and develop antibiotic resistance readily to second- and third-generation cephalosporins owing to an inducible chromosomally encoded cephalosporinase. Emergence of this form of resistance is seen frequently, when infection due to these organisms, particularly *Enterobacter cloacae*, are treated with broad-spectrum cephalosporins [19]. Pfaller et al. [20] reported that out of 230 *Enterobacter *isolates 35–50% were resistant to ceftazidime and piperacillin. In this study the resistance rates of *Enterobacter *strains to third generation cephalosporins were between 59.4–75%, whereas higher rates were recorded to aztreonam and gentamicin (both 81.2%).

Another type of commonly seen antimicrobial-resistant pathogen encountered among ICU patients is *Klebsiella pneumoniae*, which is producing extended-spectrum beta-lactamases (ESBLs). Our isolates of *K. pneumoniae *showed high resistance to broad-spectrum cephalosporins and aztreonam (72–88% and 88%, respectively).

Many studies have demonstrated that the development of antimicrobial resistance at hospital level is strongly correlated with the use of the relevant antimicrobial [21]. Data from project ICARE showed that use was significantly higher among ICU patients than non-ICU patients for third-generation cephalosporins combined, vancomycin, anti-pseudomonal penicillin, intravenous fluoroquinolones and imipenem [22]. This study supports that for each of the antimicrobial agent used at higher rates in ICU areas, there was a correspondingly higher rate of the respective resistant pathogen among ICU patients.

## Conclusion

In order to prevent the emergence and spread of antimicrobial resistant pathogens in ICU, the pattern of antimicrobial use has to be determined. A multidisciplinary approach is required to succeed in combating the problem. Hospitals should monitor antimicrobial use to determine whether specific ICUs or the entire hospital is overusing antimicrobials. These data could be used, in conjunction with other related studies, to properly interpret significant resistance patterns and choose the most appropriate antimicrobial regimens for empirical therapy.

## Methods

Data were collected from adult patients hospitalized in the surgical ICU of the Medical Faculty Hospital during one year period between January and December in 2001. Standard Centers for Disease Control and Prevention/NNIS definitions of infection were used [23]. All patients in the ICU were monitored for nosocomial infection at all body sites for a period of at least one month. Nosocomial infections were analyzed by infection site and pathogen type. Infections were considered ICU-associated if they developed in the ICU within 48 hours of admission or within 48 hours of discharge from the ICU. Bloodstream infection was reported if the patient had either two or more positive cultures drawn on separate occasions, or one positive blood culture and treatment was instituted. The data collected on each infection included the date, site of infection, age and gender of the patient. Written informed consent was obtained from all subjects prior to their inclusion in the study.

Lower respiratory tract specimens included bronchoalveolar lavage, transtracheal aspiration, and pleural fluid. Urinary tract specimens included urine and aspiration from the urinary catheter. Other types of specimens obtained from the patients were; swabs from surgical wounds, cerebrospinal fluid, pericardial fluid, peritonal fluid, synovial fluid, biopsy material, oral swab, rectal and anal swabs. All specimens were collected at the bed site, transferred to the laboratory immediately and were inoculated on proper culture media within two hours. Clinical specimens were inoculated onto 5% sheep blood agar, eosine-methylene-blue agar, Sabouraud's dextrose agar, and except the urine specimens, onto chocolate agar (Oxoid, Basingstoke, United Kingdom). Plates were incubated at 37°C in 5% CO_2 _for 18–24 hours. Methods used for confirmation of identification included examination of colonial morphology and haemolytic characteristics on appropriate agar media, Gram stain, rapid tests (catalase, oxidase, coagulase, bile solubility, spot indole, latex agglutination) and use of an automated identification system, VITEK (bioMérieux, Marcy-1'Etoile, France), which is a microbroth dilution method with two wells across pre-determined breakpoints.

Penicillin G, ampicillin, amoxicillin/clavulanate, methicillin, vancomycin, clindamycin, erythromycin, trimethoprim/sulfamethoxazol, ciprofloxacin, and tetracycline were used for antimicrobnial susceptibility testing. Susceptibility tests were performed with a commercial broth microdilution method (VITEK; bioMérieux, Marcy-1'Etoile, France) according to the manufacturers' guideline recommendations and interpretative criteria. Colonies from 18–24 hours old culture medium were used to inoculate the microdilution plates. Quality control for testing by VITEK was performed before each new lot of cards was used. Antimicrobial susceptibilities of the control microorganisms (*S. aureus *ATCC 29213, *P. aeruginosa *ATCC 27853 and *Enterococcus faecalis *ATCC 29212) were performed according to the instructions of CLSI (previously NCCLS) [24].

All data were stored and analysed using Microsoft Access and Excel. Statistical analysis was performed using the chi-squared test for independence and Fisher's exact test where appropriate. All statisticss were computed using Scientific Package for Social Sciences (SPSS) software (version 11.0; SPSS Inc., Chicago, IL).

## Authors' contributions

AB carried out the collection of clinical samples from patients, cultured and identified pathogenic microorganisms from those specimens and perfomed antimicrobial susceptibility tests of the study strains. She also designed the study and performed acquisition, analysis and interpretation of data. IB conceived of the study, and participated in its design and coordination and helped to draft the manuscript. Both authors read and approved the final version of the manuscript.

## Pre-publication history

The pre-publication history for this paper can be accessed here:


